# Increased expression of the homeostatic chemokines CCL19 and CCL21 in clinical and experimental *Rickettsia conorii* infection

**DOI:** 10.1186/1471-2334-14-70

**Published:** 2014-02-09

**Authors:** Elisabeth Astrup, Trine Ranheim, Jan K Damås, Giovanni Davì, Francesca Santilli, Mogens Jensenius, Giustina Vitale, Pål Aukrust, Juan P Olano, Kari Otterdal

**Affiliations:** 1 Institute of Clinical Medicine, Akershus University Hospital, Sykehusveien 25, Lørenskog 1478, Norway; 2 Research Institute of Internal Medicine, Sognsvannsveien 20, 0372 Oslo, Norway; 3 Section of Clinical Immunology and Infectious Diseases, Oslo University Hospital Rikshospitalet, Sognsvannsveien 20, Oslo 0372, Norway; 4 Department of Infectious Diseases, Oslo University Hospital Ullevål, Kirkeveien 166, Oslo 0450, Norway; 5 Faculty of Medicine, University of Oslo, Pb 1078 Blidern, Oslo 0316, Norway; 6 Department of Cancer Research and Molecular Medicine, Norwegian University of Science and Technology, Prinsesse Kristinas gate 3, Trondheim 7030, Norway; 7 Department of Infectious Diseases, St. Olavs Hospital, Prinsesse Kristinas gate 3, Trondheim 7030, Norway; 8 Center of Excellence on Aging, University of Chieti, Via Colle dell'Ara, 66013 Chieti, Italy; 9 Department of Clinical Medicine and Pathology, School of Medicine, University of Palermo, Palermo 90133, Italy; 10 Department of Pathology, University of Texas Medical Branch, Galveston, 301 University Blvd, TX 77555-0428, USA

**Keywords:** Inflammation, Chemokines, CCR7, R. conorii, R. africae

## Abstract

**Background:**

Based on their essential role in concerting immunological and inflammatory responses we hypothesized that the homeostatic chemokines CCL19 and CCL21 may play a pathogenic role in rickettsiae infection.

**Methods:**

Serum levels of CCL19 and CCL21 in patients with *R. africae* and *R. conorii* infection were analyzed by enzyme immunoassays. Lungs from *R. conorii* infected mice were examined for CCL19, CCL21 and CCR7 expression by immunohistochemistry.

**Results:**

We found that patients with *R. africae* infection (n = 15) and in particular those with *R. conorii* infection (n = 16) had elevated serum levels of CCL19 on admission, with a decline during follow-up. While a similar pattern was seen for CCL21 in *R. africae* infection, patients with *R. conorii* infection showed persistently increased CCL21 levels during follow-up. In experimental *R. conorii* infection, we found strong immunostaining of CCL19 and CCL21 in the lungs, particularly in individuals that had received lethal doses. Immunofluorescence showed co-localization of CCR7 to endothelial cells, macrophages and fibroblasts within the lung tissue of *R. conorii* infected mice.

**Conclusions:**

Our findings suggest that the CCL19/CCL21/CCR7 axis is up-regulated during *R. africae* and in particular during *R. conorii* infection, which may potentially contribute to the pathogenesis of these disorders.

## Background

Rickettsioses are a diverse group of acute febrile infections caused by obligate intracellular, vector-borne bacteria in the family Rickettsiales. Rickettsioses can present clinically in an array of different clinical symptoms; the most consistent being fever, myalgia, lymphadenopathy, and headache, with or without eschar and/ or maculopapular eruption [1]. The clinical spectrum of spotted fever group (SFG) rickettsioses varies in severity from mild (e.g., African tick bite fever [ATBF] caused by *Rickettsia africae [R. africae])* to potentially lethal disease with systemic multi-organ involvement, such as in some cases of Mediterranean spotted fever (MSF) caused by *R. conorii*[1].

SFG rickettsioses are characterized by infection of endothelial cells and subsequent infiltration of inflammatory mononuclear cells that results in vasculitis and increased microvascular permeability, which in severe cases, could lead to cerebral and pulmonary oedema [1,2]. The interaction between microbe and endothelial cells during SFG rickettsioses triggers an innate immune response. This response includes production of several cytokines by endothelial cells and infiltrating leukocytes, and represents both beneficial (i.e., anti-microbial) and detrimental (e.g., excessive inflammation) responses in relation to the host [1-3].

Based on their role in orchestrating lymphocyte trafficking, the chemokines CCL19 and CCL21 are commonly referred to as homeostatic chemokines. These chemokines are constitutively expressed in secondary lymphoid tissue and regulate homing of lymphocytes and dendritic cells through their common receptor CCR7 [4]. More recent studies however, have suggested that CCL19 and CCL21 may also promote inflammatory responses [5,6] and they have been implicated in the pathogenesis of various inflammatory disorders such as asthma, atherosclerosis and rheumatoid arthritis [7-9].

Based on their role in concerting immunological and inflammatory responses, we hypothesized that CCL19 and CCL21 may play a pathogenic role in rickettsiae infection. Here, we examined the expression of these chemokines in human serum samples from patients with confirmed ATBF and MSF. We also examined the expression of CCL19 and CCL21 and their common receptor CCR7 in the widely accepted murine animal model for SFG rickettsioses using C3H mice and *R. conorii* Malish 7 strain.

## Methods

### Patients and controls

The study population has previously been described [10,11]. Briefly, the *ATBF-group* (n = 15, 3 women and 12 men, 20–57 [mean age 39.2 ± 12.3 years]), was recruited from the outpatient clinic at Oslo University Hospital, Aker between January 1999 and December 2000. All patients had characteristic signs and symptoms of active ATBF (i.e., flu-like symptoms that manifested within the first 10 days after they had left rural areas in South Africa or Botswana, inoculation eschars and regional lymphadenitis with or without vesicular cutaneous rash, and aphthous stomatitis). Anti-rickettsial therapy with doxycycline (200 mg qd for 7 days) was instituted in 12 patients. For the study of *MSF* (n = 16, 7 women and 9 men, 19–74 [mean age 53.1 ± 15.6 years]), patients with confirmed MSF, at Termini Imerese Hospital Palermo, Palermo, Italy, between June and September 2005, were included. They all had characteristic signs and symptoms of active MSF (i.e., fever, eschar at the site of tick bite, and maculopapular rash). The duration of illness before diagnosis was less than 2 weeks. All MSF patients were treated with tetracycline (500 mg 4 times a day for 7 days). All MSF patients had temperature >38.5°C and severe malaise and all were hospitalized. In contrast, all the ATBF had a mild flu-like illness and none needed hospitalization. Thus, although there was no difference in disease severity within the MSF patients (all recovered during hospitalization and none had failure of one or more organs), these patient groups illustrate the difference between the mild ATBF and the more severe MSF.

Control blood samples were collected from 14 healthy travellers (3 women and 11 men, 32–66 [mean age 44.2 ± 10.7 years]) who had returned to Norway from sub-Saharan Africa during the preceding 7 days. Informed consent for participation in the study was obtained from all individuals.

All parts of the study were approved by the local ethical committee (Ethical Committee of Termini Imerese Hospital Palermo, Palermo, Italy; Regional Committee for Medical and Research Ethics, South-East Norway and Regional Committee for Medical and Research Ethics, East Norway) and conducted according to the ethical guidelines that are outlined in the Declaration of Helsinki.

### Microbiological diagnosis

All patients with ATBF fulfilled the serological criteria for *R. africae* infection: IgG plus IgM (microimmunofluorescence assay) in which titers of antibodies to *R. africae* were ≥2 dilutions higher than those to *R. conorii*; a Western-blot profile that revealed only *R. africae*-specific antibodies; or cross-adsorption studies that demonstrated that the homologous antibodies were directed against *R. africae*. All patients with MSF had seroconversion with increases in the levels of anti-*R. conorii* antibodies as assessed by enzyme-linked immunosorbent assay (ELISA) and indirect immunofluorescence.

### Blood sampling protocol

Peripheral venous blood was drawn into pyrogen-free, vacuum blood collection tubes without any additives [11] at first presentation (from days 1 to 14 after the onset of the symptoms and before the specific treatment) and at follow-up (11–21 days [ATBF] and 28–42 days [MSF] after symptom onset. Serum was stored at -80°C until analysis and samples were thawed <4 times.

### *R. conorii* infection in mice

*R. conorii* (Malish 7 strain) was obtained from the American Type Culture Collection (ATCC VR 613). For animal inoculation, rickettsiae were cultivated in embryonated chicken eggs, followed by collection of yolk sacs which were homogenized and diluted to obtain a 10% suspension in sucrose-phosphate-glutamate (SPG) buffer (0.218 M sucrose, 3.8 mM KH_2_PO_4_, 7.2 mM K_2_HPO_4_, 4.9 mM monosodium glutamic acid; pH 7.0). Rickettsiae were then purified by Renografin density gradient centrifugation and viable rickettsiae were suspended in SPG buffer. The concentration of rickettsiae from either a yolk sac or cell culture was determined by a plaque assay and quantitative real-time PCR as described below. The rickettsial stock was stored at -80°C until use.

Wild-type C3H mice were purchased from Harlan Laboratories (Indianapolis, IN) and infected between 6 to 9 weeks of age. Mice were housed in a biosafety level 3 facility at the University of Texas Medical Branch, Galveston. All experiments and procedures were approved by the University of Texas Medical Branch Animal Care and Use Committee, and mice were used according to the guidelines in the Guide for the Care and Use of Laboratory Animals. C3H mice were infected intravenously through the tail vein using a lethal dose of 3 × 10^5^ PFU (3 LD_50_ doses) or a sublethal dose of 3 × 10^4^ PFU (0.3 LD_50_ doses) of *R. conorii*. Negative control mice were inoculated with 100 μl of SPG buffer alone. Mice were monitored daily for signs of illness. Necropsies were performed at 5 days post-infection and harvested organs were fixed in 10% buffered formalin followed by paraffin embedding.

### Immunohistochemistry and immunofluorescence

Five micron sections of paraffin-embedded lungs and spleens were deparaffinised in xylene, rehydrated in alcohol series and immersed in distilled water. The sections were then treated with 0.5% H_2_0_2_, followed by high-temperature antigen retrieval in citrate buffer (pH 6), blocked with 0.5% bovine serum albumin (BSA) and incubated with primary antibodies against CCR7 (ab65851, Abcam, Cambridge, UK; 1:250), CCL19 (sc-9777, Santa Cruz Biotechnology, Dallas, TX; 1:200) or CCL21 (AF457, R&D Systems, Minneapolis, MN; 1:40) for 1 hour at room temperature. After washing, the slides were incubated for 30 minutes with peroxidase-conjugated anti-mouse Ig (Impress-Vector, Vector Laboratories, Burlingame, CA), rinsed, and developed with chromogen for immunoperoxidase staining (DAB Plus, Vector Laboratories) for 7 minutes. The sections were counterstained with hematoxylin. Omission of the primary antibody was used as negative control.

For immunofluorescence, the slides were stained with primary antibodies against CCR7 (NB110-55680, Novus Biologicals, Littleton, CO; 1:200), CD31 (DIA-310, Dianova GmbH, Hamburg, Germany; 1:50), Fibroblast Marker (sc-73355, Santa Cruz Biotechnology, San Diego, CA; 1:100), or F4/80 (MCA497RT, AbD Serotec, Kidlington, UK; 1:500) for 1 hour at room temperature and counterstained with Alexa Fluor 488 goat anti-rabbit IgG (1:500), Alexa Fluor 568 goat anti-rat IgG (1:500) or Rat on Mouse AP Polymer kit in combination with Warp Red Chromogen kit (Biocare Medical, San Francisco, CA), respectively. The slides were mounted with SlowFade Gold antifade reagent with DAPI (Life Technologies, Carlsbad, CA). Images were taken by a Nikon Eclipse E400 microscope (Tokyo, Japan).

### ELISA

Serum levels of CCL19 and CCL21 were measured by ELISAs obtained from R&D Systems. The intra- and inter-assay coefficients of variations were <10% for both ELISAs. To further minimize run-to-run variability, serial samples from a given individual were analyzed on the same tray.

### Statistical methods

Differences between groups were compared with the Mann–Whitney *U* rank-sum test for unpaired data. If more than two groups were compared, the Kruskal-Wallis test was used *a priori*. Within group differences were analyzed with Wilcoxon rank-sum test for paired data. The level of significance was set at *p* < 0.05

## Results

### Serum levels of CCL19 and CCL21 in ATBF and MSF patients

**Figure 1:**
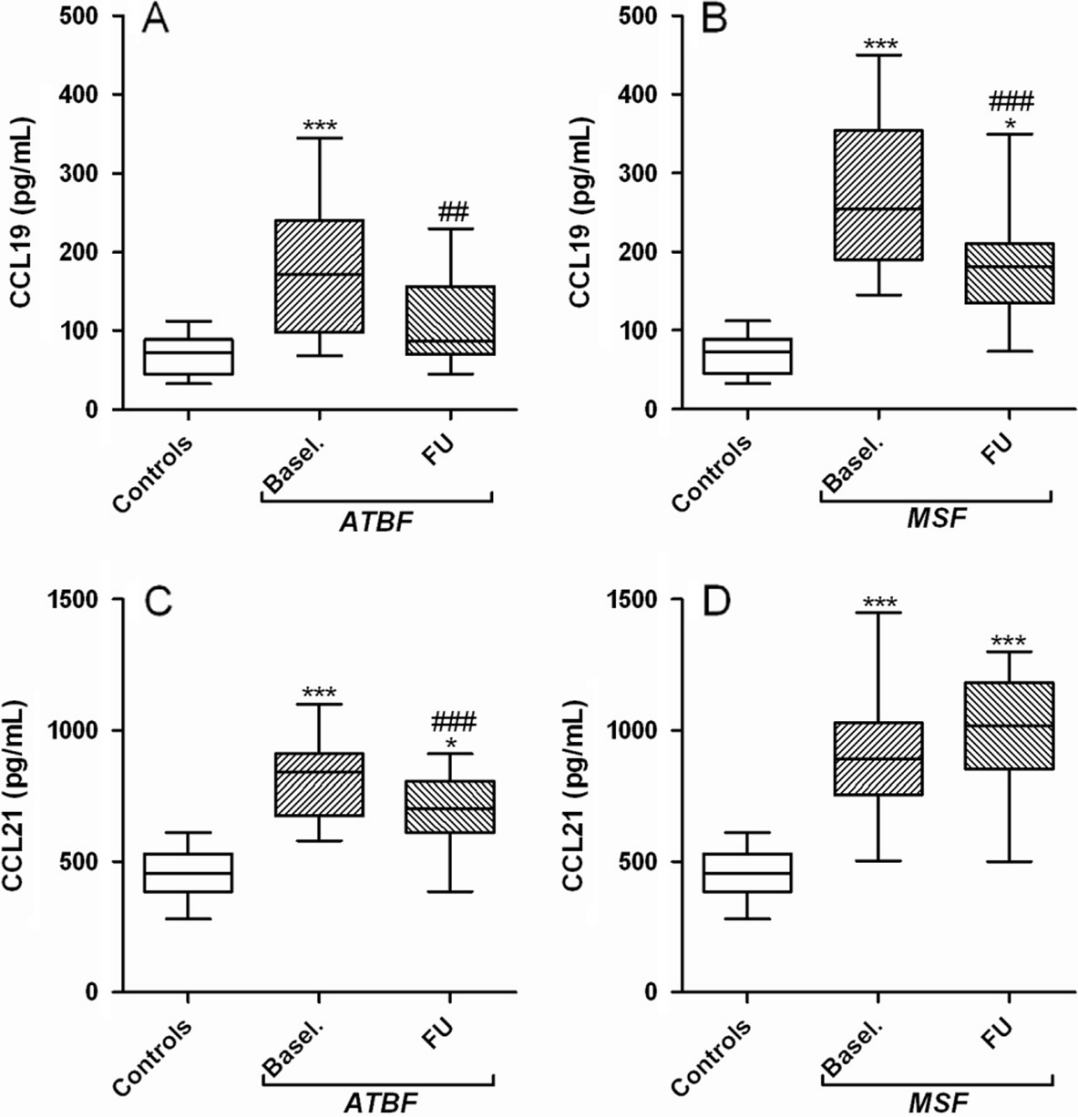
**Levels of CCL19 and CCL21 in patients and controls.** Serum levels of CCL19 **(A and B)** and CCL21 **(C and D)** is shown for 15 patients with African tick bite fever (ATBF, *R. africae)* ,16 patients with Mediterranean spotted fever (MSF, *R. conorii*) and for 14 healthy controls. Blood samples from patients were collected at admission (baseline, basel.) and follow-up (FU), 11–21 days [ATBF] and 28–42 days [MSF] after symptom onset. Twelve of the ATBF were treated with doxycycline for 7 days and all MSF patients were treated with tetracycline for 7 days following admission. Data are given as medians, 25-75 the percentiles and ranges. *p < 0.01 and ***p < 0.001 versus controls. ##p < 0.01 and ###p < 0.001 versus admission.

As seen in Figure 1A-B, patients with ATBF (n = 15) and particularly those with MSF (n = 16) had significantly raised serum levels of CCL19 at admission as compared with healthy controls (n = 14). During follow-up, after treatment with doxycycline or tetracycline in most of the patients (see Methods), there was a significant decline in CCL19 levels in both patient groups, but without full normalization (Figure 1A-B). In ATBF-patients, a similar pattern was seen for CCL21, with significantly raised levels at admission, followed by a significant decline during follow-up, without full normalization (Figure 1C). In contrast, while MSF patients also showed markedly persistently elevated CCL21 levels at admission, there was no significant changes during follow-up with a non-significant rise (p = 0.07) rather than a decline in CCL21 levels (Figure 1D).

All ATBF had a mild flu-like illness and none needed hospitalization, representing a mild form of SFG rickettsioses, whereas all MSF patients had temperature >38.5°C and severe malaise and all were hospitalized, representing a more severe form of SFG rickettsioses. At baseline CCL19, but not CCL21, tended to be higher in patients with MSF although the difference did not reach statistical significance (p = 0.08). At follow-up, there were no differences in CCL19 and CCL21 levels between ATBF and MSF patients. However, when comparing changes from baseline to follow-up, the difference between the two patient groups were statistical significant for CCL21 (p < 0.01), but not for CCL19, reflecting persistently raised CCL21 levels in patients with the more severe MSF compared to a decline during follow-up in patients with ATBF.

### CCL19, CCL21 and CCR7 expression in experimental *R. conorii* infection

**Figure 2:**
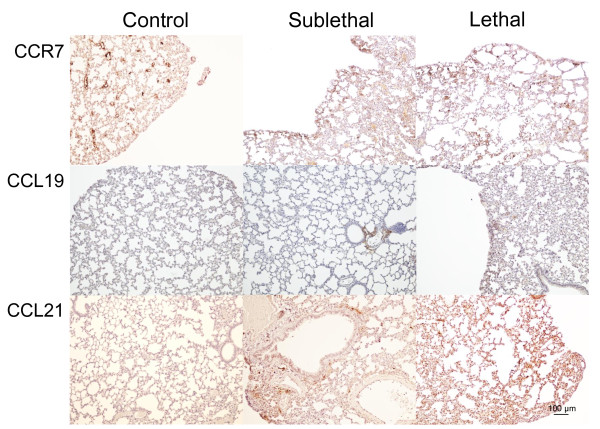
**Immunohistochemical staining of CCR7, CCL19 and CCL21 in lung tissue.** Staining of CCR7, CCL19 and CCL21 in lung tissue from control mice and from mice with sublethal and lethal doses of *R. conorii* infection is shown. Left panels show staining of lungs from controls. Middle panels and right panels show lungs from mice with sub-lethal and lethal infections, respectively. The panels show representative images from three mice in each group obtained with 10x objective. Scale bar: 100 μm.

**Figure 3:**
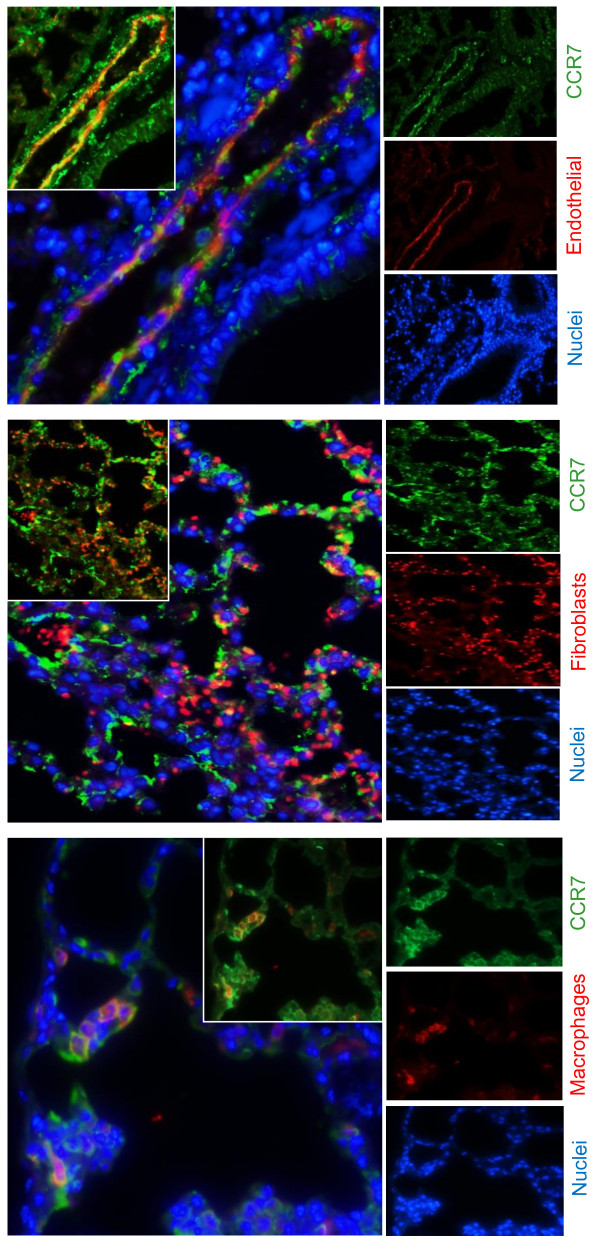
**Immunofluorescence staining of CCR7 in lung tissue.** Staining shows co-localization of CCR7 with endothelial cells, fibroblasts and macrophages in murine lungs infected with *R. conorii* (lethal infection). Lung sections were stained with CCR7 in combination with endothelial, fibroblast and macrophage cell markers. CCR7 was counterstained with Alexa Fluor 488 conjugated goat anti-rabbit IgG (green). Endothelial and fibroblast cell markers were counterstained with Alexa Fluor 568 conjugated goat anti-rat IgG (red). Macrophages were counterstained with Warp red chromogen. The nuclei were stained with Dapi (blue). Overlays are shown in the left panels. For a better visualization of co-localization of CCR7 with cell markers, inserts are overlays of only green and red staining. Similar results were obtained in 3 separate experiments.

To further study the regulation of the homeostatic chemokines CCL19 and CCL21 in SFG rickettsial infection, we examined the expression of these chemokines and their common receptor CCR7 in lungs from mice infected with sub-lethal and lethal doses of *R. conorii* as well as in uninfected mice. While only weak immunostaining of CCL19, CCL21 and CCR7 was seen in uninfected mice, strong staining was seen in *R. conorii* infected mice with particularly strong immunostaining in those that had received lethal doses (Figure 2). In general, a similar pattern was seen for CCL19, CCL21 and CCR7, but with a more prominent immunostaining for CCL21 than for CCL19. CCL19 and CCL21 interact with their common receptor CCR7, and immunofluorescence showed co-localization of CCR7 to endothelial cells, macrophages and fibroblasts within the infected lung tissue (Figure 3).

**Figure 4:**
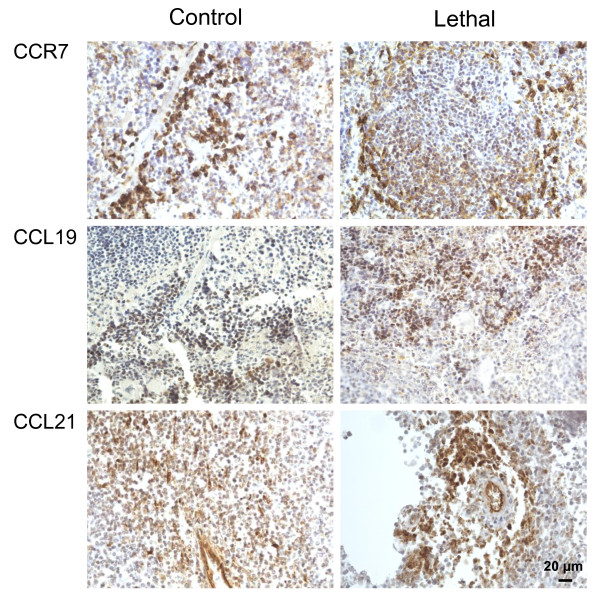
**Immunohistochemical staining of CCR7, CCL19 and CCL21 in spleen**. Panels show immunostaining of spleen from mice subjected to *R. conorii* infection. Left panels show staining of spleen from controls. Right panels show spleen from mice infected with lethal doses of *R. conorii.* Representative images from three mice in each group obtained with 40X objective. Scale bar: 20 μm.

We also examined the expression of CCL19, CCL21 and CCR7 by immunohistochemistry in secondary lymphoid organ (i.e., spleen) during experimental *R. conorii* infection (Figure 4). As expected, the immunostaining of these markers was in general stronger in the spleen as compared with the lungs. Moreover, although the staining for CCL19 and in particular CCL21 was stronger in the *R. conorii* infected mice (lethal dose) as compared with the non-infected mice, this was not seen for CCR7, and the differences were less prominent than in the lungs. Unfortunately, we lack sections from spleen in mice infected with sub-lethal doses of *R. conorii*. Whereas these findings may suggest enhanced CCL19/CCL21 expression within the spleen in *R. conorii* infected mice, the regulation of CCL19/CCL21/CCR7 seems in general to be less influenced by *R. conorii* infection in this secondary lymphoid organ than in the lungs.

## Discussion

Inflammatory chemokines such as the T-cell targeting chemokines CXCL10 and CXCL19 and the endothelial related chemokine CX3CL1 have previously been linked to the immune response against SFG rickettsial infection [12-15]. In the present study, we show that increased expression of chemokines during SFG rickettsial infection is not restricted to the inflammatory chemokines, but seems also to involve the homeostatic chemokines CCL19 and CCL21. Thus, we show increased expression of these chemokines in clinical (ATBF and MSF) and experimental (*R. conorii*) rickettsial infection. There are increased levels both systemically (ATBF and MSF), in the spleen, and in particular, in the lungs (experimental *R. conorii* infection). Within the lungs, strong immunostaining of CCR7 was seen co-localized to endothelial cells, macrophages and fibroblasts during lethal experimental *R. conorii* infection in mice.

Enhanced levels of the homeostatic chemokines CCL19 and CCL21 have previously been related to viral replication [16] and disease severity [17] in HIV infection and cytomegalovirus-induced disease in solid organ transplant recipients (CCL21) [18]. High levels have also been associated with mycobacterial infections [17,19]. In the present study, we, to the best of our knowledge, for the first time show that rickettsial infection is characterized by increased levels of CCL19 and CCL21 as well as their common receptor CCR7 being prototypical homeostatic chemokines/chemokine receptor. Moreover, in experimental murine *R. conorii* infection increased expression of CCL19 and CCR7 and in particular, CCL21 was associated with lethal infection. Also, while CCL21 declined during follow-up in ATBF, representing a mild form of SFG rickettsioses, persistently high CCL21 levels were seen in patients with the more severe MSF. In contrast to CCL21, CCL19 decreased during follow-up in both ATBF and MSF. This could be related to the fact that CCL19 and CCL21, at least to some degree, have different cellular sources. While CCL19 is produced by a wide range of cells such as circulating T cells and monocytes, the production of CCL21 seems to be restricted to stromal cells and tissue macrophages. It is therefore possible that serum levels of CCL21 in MSF patients more directly reflect the levels within the inflamed and infected tissue such as the lungs.

The pathogenic consequences of the increased expression of CCL19, CCL21 and CCR7 during *R. conorii* infection are not clear at present. Although CCR7 deficient mice were found to have compromised resistance when infected with high doses of *Mycobacterium tuberculosis*[20], high levels of CCR7 and its ligands may not necessarily be beneficial. In fact, respiratory syncytial virus infection may accelerate pulmonary disease via CCR7-related mechanisms [21]. Moreover, CCL19 and CCL21 have been found to promote an inflammatory response in peripheral blood mononuclear cells from HIV infected patients when accompanied by high viral load, a process that could be related to the inappropriate immune activation and inflammation that is seen in these patients [22]. Although it is tempting to hypothesize that the enhanced expression of CCL19 and CCL21 during *R. conorii* infection could contribute to tissue necrosis and overwhelming inflammatory responses, these issues need to be investigated in more mechanistic studies. Interestingly, Choi et al. have recently shown that *Orientia tsutsugamushi* impairs the CCL19 mediated effects on dendritic cell migration [23], and it will be of importance to examine if similar mechanisms could be seen during *R. conorii* infection.

The present study has some limitations such as the inclusion of a relatively low number of patients, and we have no data on the expression of these chemokines in relation to the degree of disease severity within the spectrum of MSF-related disease. Moreover, recent studies suggest that an MSF-like syndrome is not restricted to *R. conorii* infection, but can also be seen during infection with *R. massiliae*, *R. sibirica mongolitimona*e, *R. monacensi*[24], and the exclusion of these different species is very difficult by serological tests that were used in the present study. Also, the lack of data on the effects of CCL19 and CCL21 on rickettsia related immunity and inflammation as well as the lack of studies on experimental *R. conorii* infection in CCR7 deficient mice make it hard to draw any mechanistic conclusion from our data. However, our findings suggest that the CCL19/CCL21/CCR7 axis is up-regulated during *R. africae* and in particular during *R. conorii* infection, and future studies should examine the pathogenic consequences of these findings.

## Conclusions

In this study, we observe an increase in serum levels of the so-called homeostatic chemokines CCL19 and CCL21 in the acute phase of ATBF and MSF. The effect is more pronounced in MSF, which is also normally the more clinically severe rickettsiosis of the two, and only in ATBF did both chemokines approach levels similar to controls in the reconvalescence phase. Parallel to this, immunohistochemistry examination of lung tissue from experimental *R. conorii* infection in mice showed increased expression of both CCL19 and CCL21 as well as their common receptor CCR7, co-localized to endothelial cells, macrophages and fibroblasts, with a more profound effect in those infected with lethal dose of *R. conorii*. If these findings reflect that CCL19 and CCL21 not only carry out their homeostatic role, but also play a pathogenic role in these infections, this should be examined in forthcoming studies.

## Competing interests

The authors declare that they have no competing interests.

## Authors’ contributions

EA, PA, JKD and KO contributed to conception and design of the study. GD, FS, MJ and GV included patients and collected blood samples. JPO prepared the immunohistochemistry sections and TR performed the immunohistochemistry analyzes. EA, KO and JKD conducted the ELISA analyzes and EA and PA wrote the manuscript. All authors read, commented on and approved the final manuscript.

## Sources of financial support

Norwegian Research Council, University of Oslo, South-Eastern Norway Regional Health Authority, Invent2 Foundation.

## Pre-publication history

The pre-publication history for this paper can be accessed here:

http://www.biomedcentral.com/1471-2334/14/70/prepub
